# Conditional survival in patients with gallbladder cancer

**DOI:** 10.1186/s40880-017-0252-1

**Published:** 2017-10-30

**Authors:** Yi-Jun Kim, Kyubo Kim

**Affiliations:** Department of Radiation Oncology, Ewha Womans University College of Medicine, 1071 Anyangcheon-ro, Yangcheon-gu, Seoul, 07985 Republic of Korea

**Keywords:** Gallbladder cancer, Conditional survival, SEER

## Abstract

**Background:**

Conditional survival (CS) has been established as a clinically relevant prognostic factor for cancer survivors, and the CS in gallbladder (GB) cancer has not yet been fully evaluated. In this study, we evaluated the cancer-specific CS rate and cancer-specific survival (CSS) rate in patients with GB cancer at multiple time points and investigated prognostic factors which affect cancer-specific CS rate to provide more accurate survival information.

**Methods:**

Between 2004 and 2013, a total of 9760 patients with GB cancer were identified from the Surveillance, Epidemiology, and End Results (SEER) data. The 3-year cancer-specific CS rate was calculated using the covariate-adjusted survival function in the Cox model for each year since diagnosis, and the results were analyzed together with the adjusted CSS rates at the same time points. Cox proportional hazards regression was performed to ascertain the individual contribution of factors associated with CSS rate at diagnosis and cancer-specific CS rates at 1, 3, and 5 years after diagnosis.

**Results:**

The adjusted 5-year CSS rate was 26.1%. The adjusted 3-year cancer-specific CS rates at 1, 2, 3, 4, and 5 years after diagnosis were 55.5, 72.2, 81.5, 86.8, and 90.5%, respectively. At the time of diagnosis, age, race, histology, grade, T, N, and M categories, surgery, radiotherapy, insurance status, and marriage status were significant prognostic factors of CSS. Five years after diagnosis, however, T and M categories were significant prognostic factors for survivors (*P* = 0.007 and *P* = 0.009, respectively), whereas surgery and radiotherapy were not.

**Conclusions:**

T and M categories were significant prognostic factors even 5 years after the initial diagnosis, whereas local treatments at the time of diagnosis were not, suggesting that patients with GB cancer at high risks might need further adjuvant therapy after primary treatments. The combined analysis of CSS and cancer-specific CS rates offered more accurate survival information for patients who have already survived a certain period of time after diagnosis.

## Introduction

Although gallbladder (GB) cancer is not a common malignancy with approximately 5000 new cases per year in the United States, it is the most common biliary tract cancer and the fifth most common gastrointestinal malignancy [1]. The prognosis of GB cancer is poor, with a 5-year overall survival (OS) rate of 5%–17% [2–5]. North Indians [6] and South American Indians [7] have a high incidence of GB cancer with a worse prognosis, as the 5-year survival rate is reported to be less than 10%. However, more aggressive surgery and the use of adjuvant therapy have improved survival outcomes over the last few decades [8–11].

Prognostic estimation for cancer patients is usually performed using the cancer-specific survival (CSS) measurement. As the CSS rate is defined as the survival probability from diagnosis to a specific time point, CSS underestimates the actual survival rates of the cancer survivors who have already survived a certain period. This underestimation is prominent in cancer patients with poor CSS such as GB cancer patients.

Cancer-specific conditional survival (CS) rate is defined as the probability that a cancer patient will survive some additional number of years, given the condition that the patient has already survived for a certain number of years. In actual clinical practice, the cancer-specific CS rate is informative for patients and their clinicians because the cancer-specific CS rate applies the condition that the patient is still alive [12]. Incorporating information on alive status generates more relevant survival estimation (CS rate) than the classical CSS rate [13]. Therefore, the use of cancer-specific CS rate is meaningful during patient counseling.

Furthermore, the cancer-specific CS rate can be used as a surrogate for cure rate. If the cancer-specific CS rate reaches a plateau (i.e., ceiling) at a certain time point, a patient who survives to the time point with no evidence of disease can be considered to be cured [14]. Analyzing the cancer-specific CS rate of GB cancer may provide quantitative insight into the curability of GB cancer.

In this study, we calculated the cancer-specific CS rate of GB cancer using the Surveillance, Epidemiology, and End Results (SEER) database between 2004 and 2013. The over-time changes of prognostic significance of patient, tumor, and treatment-related factors were analyzed.

## Methods and materials

### Patient population

The SEER 18-registry dataset (a set of 18 population-based regional cancer registries) was used in this study. Patients who were pathologically or clinically diagnosed with primary GB cancer (ICD-0-3 code 23.9/WHO 2008) between 2004 and 2013 were identified. The patients with unknown survival time were excluded. Tumors were classified according to the 7th edition of the American Joint Committee on Cancer (AJCC) staging manual [15].

### Statistical analysis

The formula of cancer-specific CS rate is as follows [12]; CSS(*t*) is the *t*-year CSS rate. Cancer-specific CS(*y*|*x*) is the additional *y*-year CSS rate, given the condition that the person has already survived *x* years.

$$cancer-specific\;CS\left( {y|x} \right) = \frac{CSS(x + y)}{CSS(x)}$$

For example, to calculate the 3-year cancer-specific CS rate for a patient who has already survived 2 years (*x* = 2, *y* = 3), the 5-year CSS rate, CSS(2 + 3), is divided by the 2-year CSS rate, CSS(2). Suppose that there were 100 patients diagnosed with GB cancer. Among them, 50 patients have survived from the cancer for 2 years [CSS(2) = 0.5], and 20 patients have survived for 5 years [CSS(5) = 0.2]. In that case, the 3-year cancer-specific CS rate at 2 years after diagnosis is 0.4 (0.2/0.5).

$$cancer-specific\;CS\left( {3|2} \right) = \frac{CSS(2 + 3)}{CSS(2)} = \frac{{\frac{20}{100}}}{{\frac{50}{100}}} = \frac{20}{50} = 0.4$$

In this formula, the initial settings of 100 patients and the 2-year time interval are eliminated. Cancer-specific CS(3|2) can be defined as the 3-year CSS rate of the selective patients who survived for 2 years (*n* = 50) calculated by using newly formatted survival time (subtraction of 2 years from the initial survival time).

With this concept, the 3-year cancer-specific CS rates for the patients who survived for *x* years were computed by following procedures: (1) selection of *x*-year survivors; (2) subtraction of *x* years from the initial survival time; and (3) calculation of a 3-year CSS rate for the survivors using the modified survival time.

The Kaplan–Meier method was used to estimate the CSS and cancer-specific CS rates. To calculate 95% confidence intervals (CIs) of the CSS and cancer-specific CS rates, the log–log transformation of survival was used [16].

Multivariate Cox proportional-hazards regression was performed to evaluate the hazard of CSS rate at the time of diagnosis and cancer-specific CS rates for multiple survival periods (1, 3, and 5 years after diagnosis). For instance, to compute the cancer-specific CS rate at 1 year after diagnosis, 1-year survivors were selected. After subtraction of 12 months from their survival time, a multivariate analysis was performed. Incorporated variables for the analysis at diagnosis were demographic (age at diagnosis, sex, race, marital status, insurance status), tumor (histology, grade, T, N, and M categories), and treatment-related factors (surgical extent, radiotherapy). Only the variables which were prognostic with *P* value less than 0.1 in the analysis of the previous period were selected and incorporated in the next period’s multivariate analysis sequentially. The multivariate Cox proportional hazards regression was performed using SPSS version 22.0 (SPSS Inc., Chicago, IL, USA).

At the same time, considering the potential influence of covariates on the survival at each time point, the covariate-adjusted survival function in the Cox model was used to estimate the adjusted CSS and cancer-specific CS rates. Specifically, the CSS and cancer-specific CS rates calculated with an adjustment for age, sex, race, histology, grade, T, N, and M categories, surgery, radiotherapy, insurance, and marital status. The log–log-based point-wise CIs were obtained for the adjusted CSS and cancer-specific CS rates [17]. For example, to calculate the adjusted 3-year cancer-specific CS rate at 1 year after diagnosis, the patients who have survived 12 months were selected and these 12 months were subtracted from the survival times of the survivors. Subsequent analysis was performed using the Cox regression while incorporating all variables. Given the estimated coefficients from the Cox model, a covariate-adjusted survival function estimate was performed, and the estimated survival rate at 36 months (that is, 48 months from diagnosis) was obtained as the adjusted 3-year cancer-specific CS rate at 1 year after diagnosis.

For subgroup analyses, the variables, which were found to be significant prognostic factors in the multivariate analyses, were selected to divide patients into multiple risk groups. The adjusted CSS rates and 3-year cancer-specific CS rates at 1, 2, 3, 4, and 5 years after diagnosis were calculated and compared among risk groups.

All calculations of unadjusted or adjusted CSS and cancer-specific CS rates were carried out using STATA/MP (ver. 14.2; StataCorp LP, College Station, TX, USA).

## Results

### Demographic and clinicopathologic characteristics

A total of 9760 patients diagnosed with GB cancer between 2004 and 2013 were included in our analyses. The number of patients who were still alive was 3232, 1178, and 612 at 1, 3, and 5 years after diagnosis, respectively. Table 1 shows the characteristics of the patients at diagnosis as well as at 1, 3, and 5 years after diagnosis.

**Table 1:** Characteristics of patients with gallbladder cancer at the time of diagnosis and survivor characteristics at 1, 3, and 5 years after diagnosis

Characteristic	At diagnosis (*n* = 9760)	Time after diagnosis		
		1 year (*n* = 3232)	3 years (*n* = 1178)	5 years (*n* = 612)
Age at diagnosis (years)				
< 65	3056 (31.3)	1223 (37.8)	470 (39.9)	251 (41.0)
65–79	3975 (40.7)	1339 (41.4)	493 (41.9)	261 (42.6)
≥ 80	2729 (28.0)	670 (20.7)	215 (18.3)	100 (16.3)
Sex				
Men	3012 (30.9)	956 (29.4)	320 (27.2)	158 (25.8)
Women	6748 (69.1)	2276 (70.4)	858 (72.8)	454 (74.2)
Race				
White	7528 (77.1)	2506 (77.5)	908 (77.1)	473 (77.3)
Black	1163 (11.9)	370 (11.4)	128 (10.9)	64 (10.5)
Others	1040 (10.7)	347 (10.7)	139 (11.8)	73 (11.9)
Unknown	29 (0.3)	9 (0.3)	3 (0.3)	2 (0.3)
Histology				
Non-papillary	9415 (96.5)	3007 (93.0)	1059 (89.9)	547 (89.4)
Papillary	345 (3.5)	225 (7.0)	119 (10.1)	65 (10.6)
Grade				
Grade 1 or 2	3648 (37.4)	1821 (56.3)	716 (60.8)	372 (60.8)
Grade 3 or 4	2874 (29.4)	790 (24.4)	243 (20.6)	121 (19.8)
Unknown	3238 (33.2)	621 (19.2)	219 (18.6)	119 (19.4)
T category				
T1	1520 (15.6)	817 (25.3)	433 (36.8)	257 (42.0)
T2	2354 (24.1)	1276 (39.5)	504 (42.8)	241 (39.4)
T3–4	4492 (46.0)	979 (30.3)	212 (18.0)	99 (16.2)
Unknown	1394 (14.3)	160 (5.0)	29 (2.5)	15 (2.5)
N category				
N0	5521 (56.6)	2220 (68.7)	927 (78.7)	495 (80.9)
N1–2	2623 (26.9)	786 (24.3)	188 (16.0)	82 (13.4)
Unknown	1616 (16.6)	226 (7.0)	63 (5.3)	35 (5.7)
M category				
M0	5594 (57.3)	2665 (82.5)	1085 (92.1)	568 (92.8)
M1	3266 (33.5)	408 (12.6)	40 (3.4)	16 (2.6)
Unknown	900 (9.2)	159 (4.9)	53 (4.5)	28 (4.6)
Surgery				
No	3393 (34.8)	348 (10.8)	55 (4.7)	32 (5.2)
Yes	6321 (64.8)	2873 (88.9)	1119 (95.0)	578 (94.4)
Unknown	46 (0.5)	11 (0.3)	4 (0.3)	2 (0.3)
Radiotherapy				
No	8329 (85.3)	2483 (76.8)	949 (80.6)	505 (82.5)
Yes	1254 (12.8)	687 (21.3)	215 (18.3)	100 (16.3)
Unknown	177 (1.8)	62 (1.9)	14 (1.2)	7 (1.1)
Insurance				
No	1484 (15.2)	436 (13.5)	134 (11.4)	47 (7.7)
Yes	5311 (54.4)	1769 (54.7)	547 (46.4)	204 (33.3)
Unknown	2965 (30.4)	1027 (31.8)	497 (42.2)	361 (59.0)
Marriage				
No	4610 (47.2)	1365 (42.2)	480 (40.7)	237 (38.7)
Yes	4689 (48.0)	1723 (53.3)	651 (55.3)	346 (56.5)
Unknown	461 (4.7)	144 (4.5)	47 (4.0)	29 (4.7)

All values are presented as number of cases followed by percentage in parentheses

At diagnosis, the majority of patients were women (*n* = 6748, 69.1%) and white (*n* = 7528, 77.1%). A considerable proportion of patients had high-risk tumors with advanced T category (T3 or T4, *n* = 4492, 46.0%), lymph node involvement (*n* = 2623, 26.9%), and distant metastasis (*n* = 3266, 33.5%). A majority of patients (*n* = 6321, 64.8%) received surgical treatment at primary site. Radiotherapy was administered in 1254 (12.8%) patients.

### Trends of CSS and adjusted CSS rates

**Fig. 1:**
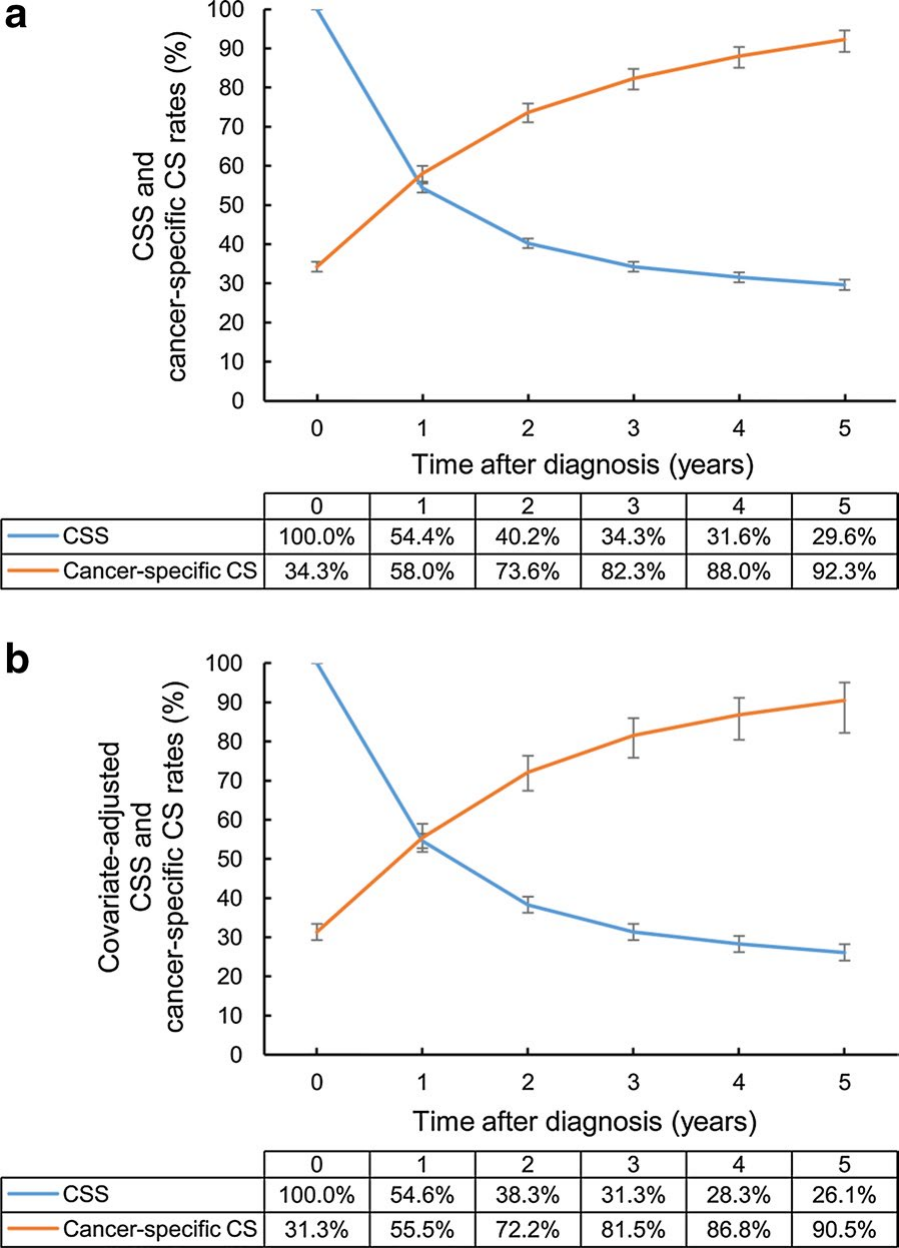
CSS and 3-year cancer-specific CS rates of gallbladder cancer patients: **a** unadjusted (Kaplan–Meier) and **b** covariate-adjusted (Cox model) CSS and CS rates. Error bars indicate 95% confidence intervals (CIs). *CSS* cancer-specific survival, *CS* conditional survival

The overall CSS rate was 54.4, 40.2, 34.3, 31.6, and 29.6% at 1, 2, 3, 4, and 5 years, respectively. The covariate-adjusted CSS rate showed a similar trend as the unadjusted CSS rate, although that was slightly lower (54.6, 38.3, 31.3, 28.3, and 26.1% at 1, 2, 3, 4, and 5 years, respectively) (Fig. 1).

### Trends of CS and adjusted CS rates

While the CSS rate decreased over time, the 3-year cancer-specific CS rate increased gradually in each successive year. The increased 3-year cancer-specific CS rate approached a plateau phase (82.3, 88.0, and 92.3% at 3, 4, and 5 years, respectively), suggesting that the majority of survivors at 5 years after diagnosis achieved stable cures of their GB cancer (Fig. 1). Even after covariate adjustment, the cancer-specific CS rates increased over time to reach a plateau. Although the rates were slightly lower than the unadjusted rates, the adjusted cancer-specific CS rate reached to 90.5% at 5 years after diagnosis.

### Factors associated with CSS and cancer-specific CS rates

Upon multivariate analysis at the time of diagnosis, patients who were young, white, had papillary histology, had low grade tumors, low T, N, and M categories, underwent surgery and radiotherapy, were insured, and were married had significantly higher CSS rates than the controls (Table 2). At 1 year after diagnosis, age 80 years or more (*P* = 0.007), black race (*P* = 0.040), high grade (*P* = 0.043), high T, N, and M categories (*P* < 0.001 for all) were identified as significantly adverse predictors, and papillary histology (*P* = 0.003), surgery (*P* = 0.012), and radiotherapy (*P* = 0.015) were significantly favorable predictors. At 3 years after diagnosis, T3–4 and M1 diseases continued to predict low cancer-specific CS rate for survivors (*P* < 0.001 and *P* = 0.038, respectively). On multivariate analysis incorporating age, T and M categories, and surgery at 5 years after diagnosis, we found T category (for T2 disease, hazard ratio [HR] 1.346 and 95% CI 0.567–3.199, *P* = 0.501; for T3–4 disease, HR 3.388 and 95% CI 1.400–8.196; *P* = 0.007) and M category (for M1, HR 4.620 and 95% CI 1.476–14.457; *P* = 0.009) to be persistently significant prognostic factors.

**Table 2:** Cox proportional hazards regression analyses of cancer-specific survival at the time of diagnosis and cancer-specific conditional survival for gallbladder cancer survivors at 1, 3, and 5 years after diagnosis

Characteristics	At diagnosis (*n* = 9760)				1 year after diagnosis (*n* = 3232)				3 years after diagnosis (*n* = 1178)				5 years after diagnosis (*n* = 612)			
	Patients	CSD	HR (95% CI)	*P* value	Survivors	CSCD	HR (95% CI)	*P* value	Survivors	CSCD	HR (95% CI)	*P* value	Survivors	CSCD	HR (95% CI)	*P* value
Age at diagnosis (years)																
< 65	3056	1760 (57.6)	Reference		1223	477 (39.0)	Reference		470	63 (13.4)	Reference		251	15 (6.0)	Reference	
65–79	3975	2177 (54.8)	1.178 (1.104–1.256)	< 0.001	1339	472 (35.3)	1.038 (0.912–1.182)	0.574	493	71 (14.4)	1.117 (0.789–1.581)	0.534	261	15 (5.7)	0.971 (0.472–1.998)	0.937
≥ 80	2729	1495 (54.8)	1.584 (1.471–1.706)	< 0.001	670	213 (31.8)	1.271 (1.068–1.513)	0.007	215	32 (14.9)	1.504 (0.967–2.338)	0.070	100	4 (4.0)	0.885 (0.290–2.698)	0.830
Sex																
Men	3012	1633 (54.2)	Reference		956	349 (36.5)	NA		320	47 (14.7)	NA		158	11 (7.0)	NA	
Women	6748	3799 (56.3)	0.988 (0.931–1.049)	0.689	2276	813 (35.7)			858	119 (13.9)			454	23 (5.1)		
Race																
White	7528	4148 (55.1)	Reference		2506	886 (35.4)	Reference		908	134 (14.8)	Reference		473	28 (5.9)	NA	
Black	1163	683 (58.7)	1.095 (1.009–1.189)	0.030	370	151 (40.8)	1.202 (1.009–1.432)	0.040	128	15 (11.7)	0.782 (0.455–1.346)	0.375	64	5 (7.8)		
Others	1040	596 (57.3)	0.997 (0.914–1.087)	0.945	347	125 (36.0)	1.005 (0.831–1.215)	0.958	139	17 (12.2)	0.861 (0.515–1.441)	0.569	73	1 (1.4)		
Unknown	29	5 (17.2)	0.374 (0.156–0.900)	0.028	9	0 (0.0)	NA (NA)	0.873	3	0 (0.0)	NA (NA)	0.970	2	0 (0.0)		
Histology																
Non-papillary	9415	5343 (56.7)	Reference		3007	1120 (37.2)	Reference		1059	156 (14.7)	Reference		547	33 (6.0)	NA	
Papillary	345	89 (25.8)	0.550 (0.444–0.680)	< 0.001	225	42 (18.7)	0.622 (0.454–0.851)	0.003	119	10 (8.4)	0.792 (0.411–1.527)	0.486	65	1 (1.5)		
Grade																
Grade 1–2	3648	1549 (42.5)	Reference		1821	580 (31.9)	Reference		716	105 (14.7)	Reference		372	25 (6.7)	NA	
Grade 3–4	2874	1760 (61.2)	1.523 (1.419–1.634)	< 0.001	790	353 (44.7)	1.152 (1.004–1.322)	0.043	243	40 (16.5)	0.744 (0.506–1.093)	0.132	121	6 (5.0)		
Unknown	3238	2123 (65.6)	1.144 (1.052–1.244)	0.002	621	229 (36.9)	0.776 (0.639–0.942)	0.010	219	21 (9.6)	0.703 (0.409–1.206)	0.200	119	3 (2.5)		
T category																
T1	1520	525 (34.5)	Reference		817	160 (19.6)	Reference		433	34 (7.9)	Reference		257	9 (3.5)	Reference	
T2	2354	870 (37.0)	1.153 (1.032–1.289)	0.012	1276	361 (28.3)	1.358 (1.118–1.649)	< 0.002	504	61 (12.1)	1.446 (0.931–2.247)	0.101	241	12 (5.0)	1.346 (0.567–3.199)	0.501
T3–4	4492	3033 (67.5)	2.356 (2.133–2.602)	< 0.001	979	551 (56.3)	2.916 (2.405–3.537)	< 0.001	212	62 (29.2)	3.948 (2.486–6.270)	< 0.001	99	13 (13.1)	3.388 (1.400–8.196)	0.007
Unknown	1394	1004 (72.0)	1.897 (1.676–2.148)	< 0.001	160	90 (56.3)	2.629 (1.913–3.613)	< 0.001	29	9 (31.0)	7.250 (2.871–18.309)	< 0.001	15	0 (0.0)	NA (NA)	0.980
N category																
N0	5521	2601 (47.1)	Reference		2220	646 (29.1)	Reference		927	114 (12.3)	Reference		495	20 (4.0)	NA	
N1–2	2623	1683 (64.2)	1.213 (1.137–1.294)	< 0.001	786	407 (51.8)	1.493 (1.301–1.714)	< 0.001	188	41 (21.8)	1.272 (0.845–1.915)	0.248	82	12 (14.6)		
Unknown	1616	1148 (71.0)	1.277 (1.173–1.392)	< 0.001	226	109 (48.2)	1.230 (0.947–1.598)	0.120	63	11 (17.5)	1.088 (0.429–2.763)	0.858	35	2 (5.7)		
M category																
M0	5594	2427 (43.4)	Reference		2665	831 (31.2)	Reference		1085	143 (13.2)	Reference		568	30 (5.3)	Reference	
M1	3266	2385 (73.0)	2.050 (1.912–2.199)	< 0.001	408	258 (63.2)	2.346 (1.987–2.769)	< 0.001	40	12 (30.0)	2.022 (1.040–3.932)	0.038	16	4 (25.0)	4.620 (1.476–14.457)	0.009
Unknown	900	620 (68.9)	1.417 (1.274–1.575)	< 0.001	159	73 (45.9)	1.051 (0.784–1.409)	0.741	53	11 (20.8)	1.093 (0.447–2.675)	0.845	28	0 (0.0)	NA (NA)	0.976
Surgery																
No	3393	2427 (71.5)	Reference		348	190 (54.6)	Reference		55	4 (7.3)	Reference		32	1 (3.1)	Reference	
Yes	6321	2970 (47.0)	0.576 (0.532–0.624)	< 0.001	2873	968 (33.7)	0.754 (0.605–0.940)	0.012	1119	162 (14.5)	4.403 (1.466–13.219)	0.008	578	33 (5.7)	3.000 (0.372–24.190)	0.302
Unknown	46	35 (76.1)	0.980 (0.684–1.404)	0.913	11	4 (36.4)	0.841 (0.299–2.365)	0.742	4	0 (0.0)	NA (NA)	0.969	2	0 (0.0)	NA (NA)	0.994
Radiotherapy																
No	8329	4694 (56.4)	Reference		2483	829 (33.4)	Reference		949	120 (12.6)	Reference		505	25 (5.0)	NA	
Yes	1254	641 (51.1)	0.740 (0.679–0.808)	< 0.001	687	314 (45.7)	1.195 (1.035–1.379)	0.015	215	45 (20.9)	1.378 (0.933–2.035)	0.107	100	9 (9.0)		
Unknown	177	97 (54.8)	0.876 (0.707–1.085)	0.225	62	19 (30.6)	0.897 (0.558–1.441)	0.653	14	1 (7.1)	0.583 (0.080–4.233)	0.594	7	0 (0.0)		
Insurance																
No	1484	804 (54.2)	Reference		436	135 (31.0)	Reference		134	13 (9.7)	NA		47	0 (0.0)	NA	
Yes	5311	2766 (52.1)	0.891 (0.822–0.966)	0.005	1769	586 (33.1)	1.049 (0.867–1.269)	0.625	547	53 (9.7)			204	9 (4.4)		
Unknown	2965	1862 (62.8)	1.061 (0.975–1.155)	0.170	1027	441 (42.9)	1.154 (0.948–1.405)	0.154	497	100 (20.1)			361	25 (6.9)		
Marriage																
No	4610	2603 (56.5)	Reference		1365	454 (33.3)	Reference		480	70 (14.6)	NA		237	15 (6.3)	NA	
Yes	4689	2591 (55.3)	0.904 (0.852–0.958)	0.001	1723	662 (38.4)	1.093 (0.965–1.239)	0.163	651	91 (14.0)			346	19 (5.5)		
Unknown	461	238 (51.6)	0.905 (0.792–1.035)	0.144	144	46 (31.9)	1.011 (0.746–1.371)	0.942	47	5 (10.6)			29	0 (0.0)		

Patients at the time of diagnosis and survivors at 1, 3, and 5 years after diagnosis are presented as number of cases. CSD and CSCD are presented as number of cases followed by percentage in parentheses

*CSD* cancer-specific death, *CSCD* cancer-specific conditional death, *HR* hazard ratio, *CI* confidence interval, *NA* not applicable

### Subgroup analysis of CSS and cancer-specific CS rates

The adjusted 5-year CSS rates of patients with T1, T2, and T3–4 disease were 56.8, 31.6, and 9.9%, respectively. The adjusted 3-year cancer-specific CS rate for patients with T3–4 disease showed the greatest improvement from 13.9% at 1 year after diagnosis to 84.4% at 5 years after diagnosis. However, the significant inferiority of the 3-year cancer-specific CS rate continued at 3 years after diagnosis. The 3-year cancer-specific CS rates at 5 years for patients with T1, T2, and T3–4 disease were 94.0, 93.0, and 84.4%, respectively, suggesting that the 5-year survivors with T1–2 diseases achieved stable cures of their disease.

**Fig. 2:**
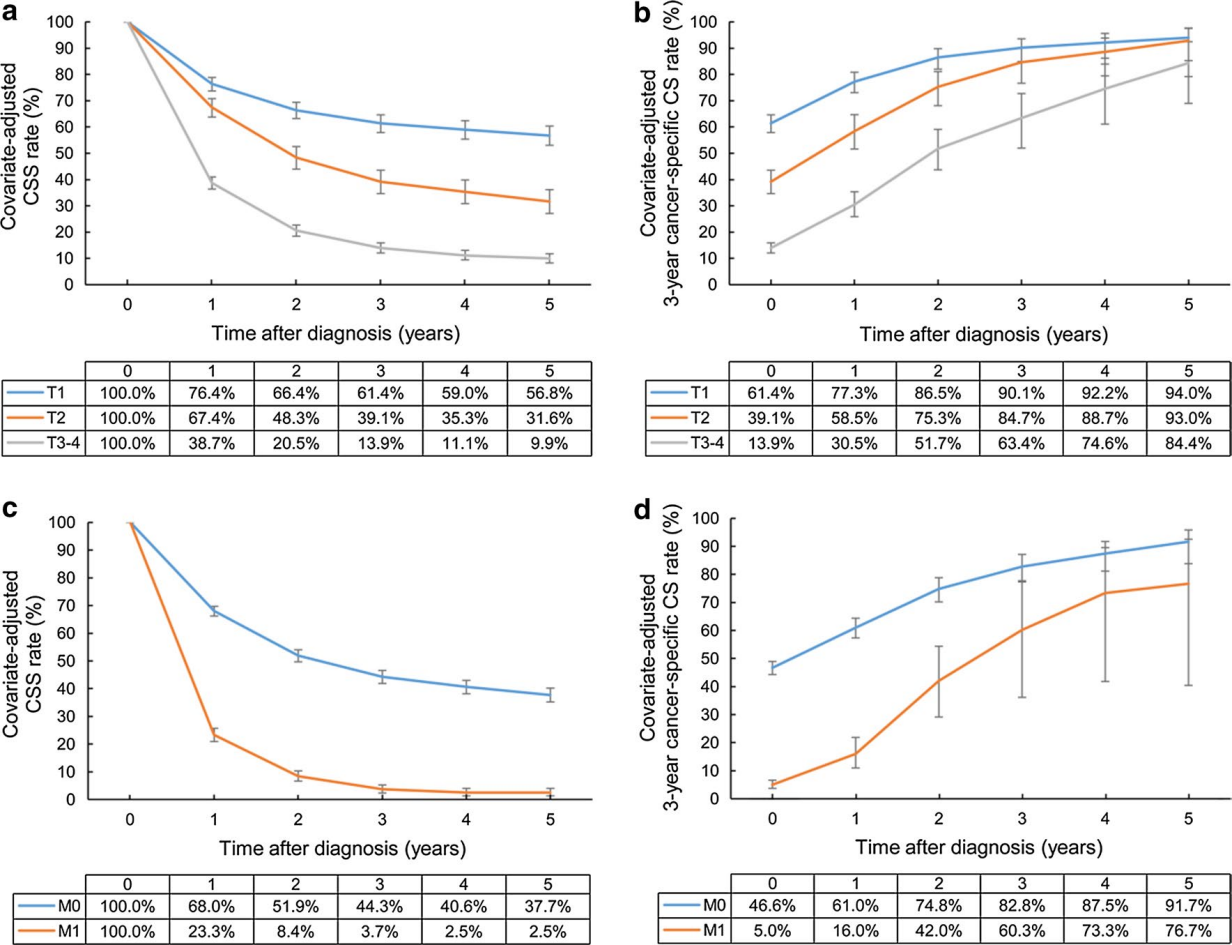
Covariate-adjusted (Cox model) CSS and 3-year cancer-specific CS rates of gallbladder cancer patients according to T and M categories: **a** CSS rates according to T category; **b** cancer-specific CS rates according to T category; **c** CSS rates according to M category; **d** cancer-specific CS rates according to M category. Error bars indicate 95% CIs. *CSS* cancer-specific survival, *CS* conditional survival

The adjusted 5-year CSS rate of patients with distant metastasis (M1 disease) at time of diagnosis was only 2.5%. The improvement of 3-year cancer-specific CS rate was decelerated and eventually ceased at 76.7% at 5 years after diagnosis, indicating that patients with M1 disease at diagnosis still experience disease progression despite surviving 5 years (Fig. 2).

**Fig. 3:**
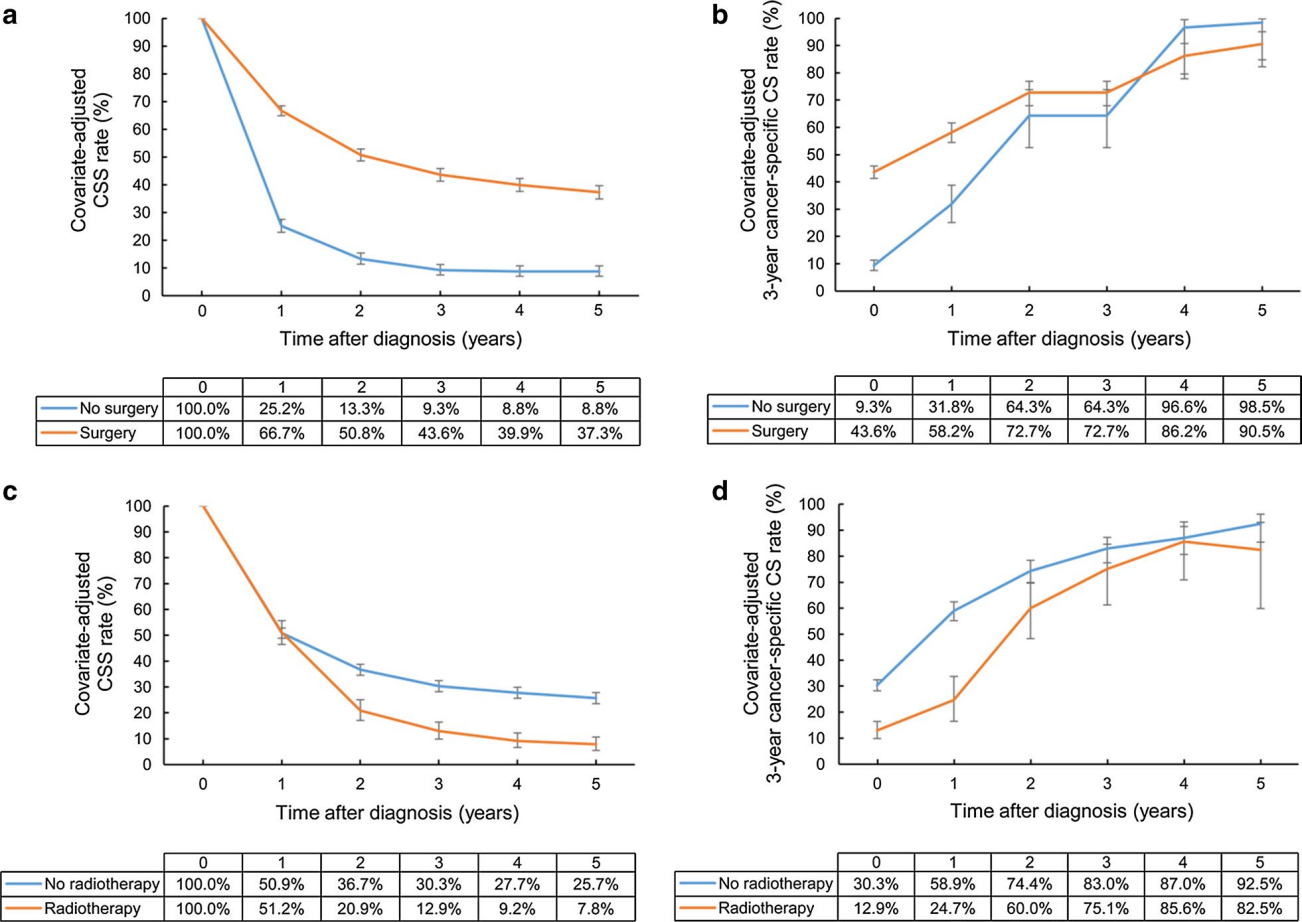
Covariate-adjusted (Cox model) CSS and 3-year cancer-specific CS rates of gallbladder cancer patients according to treatments: **a** CSS rates according to surgery; **b** cancer-specific CS rates according to surgery; **c** CSS rates according to radiotherapy; **d** cancer-specific CS rates according to radiotherapy. Error bars indicate 95% CIs. *CSS* cancer-specific survival, *CS* conditional survival

Surgery increased the 5-year CSS rate from 8.8% to 37.3%, with a 3-year cancer-specific CS rate of 90.5% at 5 years after diagnosis. However, radiotherapy did not show any CSS and cancer-specific CS benefits. The patients who had low cancer-specific CS rates at an early period in the radiotherapy group were prone to have a low CSS rate at the late period. At 5 years after diagnosis, the CSS rates were 25.7% and 7.8% in the non-radiotherapy group and radiotherapy group, respectively. The adjusted 3-year cancer-specific CS rate at 5 years after diagnosis in the radiotherapy group was 82.5% (Fig. 3).

In subgroup analyses for patients treated with surgery, T and M categories were still significant prognostic factors. The 5-year CSS rates of patients with T1, T2, and T3–4 diseases were 65.7, 48.9, and 14.9%, respectively, and the patients with T1 and T2 diseases had 3-year cancer-specific CS rates of higher than 90% at 5 years after diagnosis (94.2% and 92.5%, respectively), whereas those with T3–4 diseases did not (78.6%). Even after surgery, patients with M1 disease showed a low 5-year CSS rate (7.1% vs. 45.5%) and 3-year cancer-specific CS rate (42.8% vs. 91.6%) at 5 years after diagnosis compared with patients with M0 disease.

**Fig. 4:**
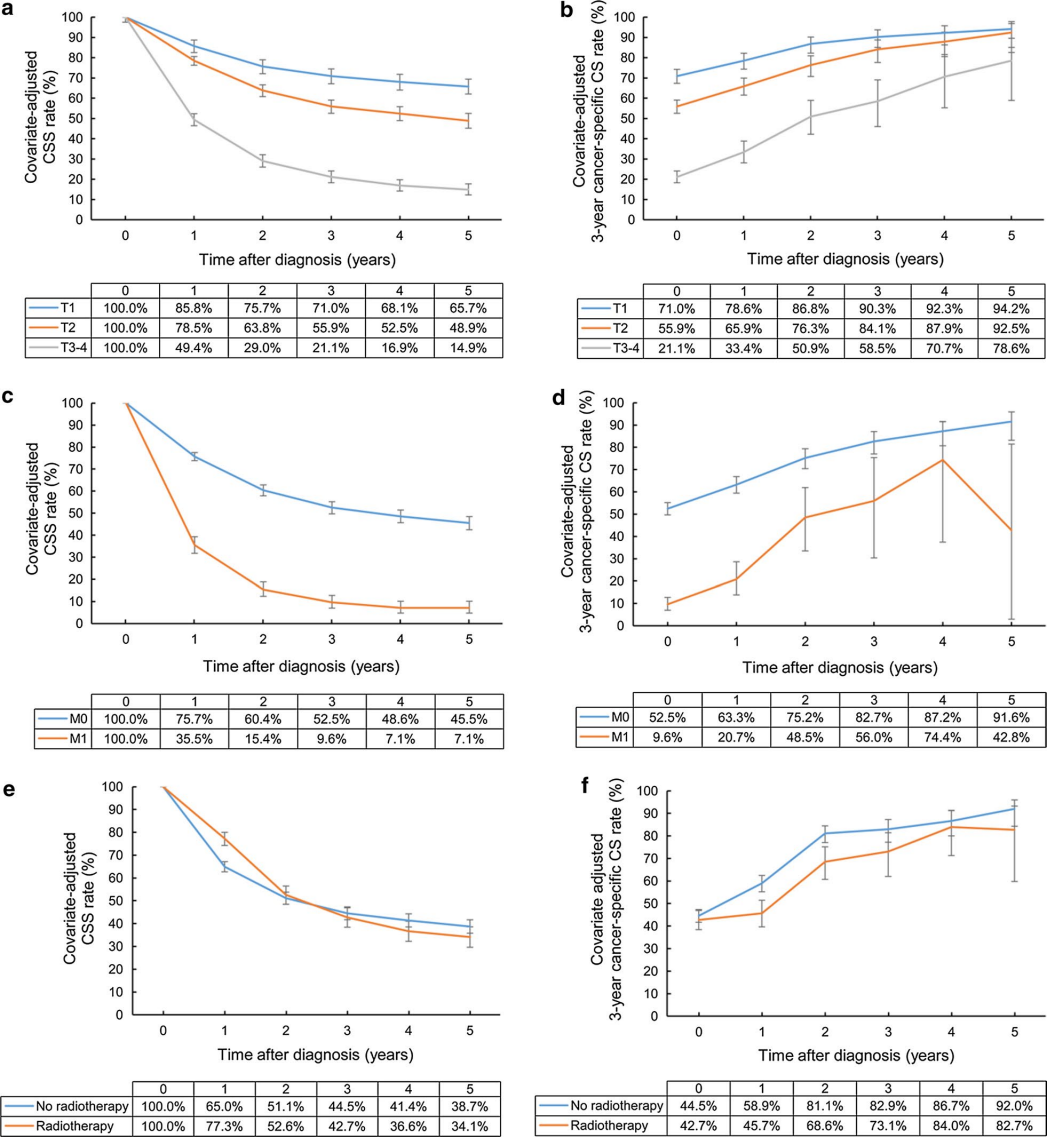
Covariate-adjusted (Cox model) CSS and 3-year cancer-specific CS rates of gallbladder cancer patients treated with surgery: **a** CSS rates according to T category; **b** cancer-specific CS rates according to T category; **c** CSS rates according to M category; **d** cancer-specific CS rates according to M category; **e** rates CSS according to radiotherapy; **f** cancer-specific CS rates according to radiotherapy. Error bars indicate 95% CIs. *CSS* cancer-specific survival, *CS* conditional survival

When radiotherapy was administered after surgery, the adjusted CSS rate at 1 year after diagnosis increased from 65.0% to 77.3%. However, radiotherapy decreased the 3-year cancer-specific CS rate at an early period, resulting in a 5-year CSS rate of 34.1% in the radiotherapy group, which was similar to 38.7% in the non-radiotherapy group (Fig. 4).

## Discussion

In the present study, we found that the 3-year cancer-specific CS rate of GB cancer patients increased over time, resulting in the covariate-adjusted 3-year cancer-specific CS rate of 90.5% at 5 years after diagnosis. The covariate-adjusted 5-year CSS rate was 26.1%. Therefore, 26.1% of GB cancer patients who survived 5 years can be expected to survive 3 more years with 90.5% of probability. T and M categories were significant prognostic factors at the time of diagnosis and their prognostic effects persisted until 5 years after diagnosis. However, local treatments at the time of diagnosis including surgery and radiotherapy were not prognostic factors at 5 years after diagnosis. The combined information of CSS and cancer-specific CS rates allowed more efficient prognostic and predictive analyses.

The CS rate is higher than the CSS rate estimated at the time of diagnosis. If a patient is still alive 2 years after diagnosis, the 3-year CS rate at 2 years after diagnosis is higher than the 5-year CSS rate, because the 5-year CSS rate includes the probability of patients who died within 2 years after diagnosis. For patients who survive, the CS rate can offer more accurate information regarding survival estimation compared with the traditionally used CSS rate. The cancer-specific CS rate is usually higher than the CSS rate even in patients who are alive with disease, and the difference between both rates is even more distinct for patients with poor prognosis cancers [18]. Therefore, the cancer-specific CS rate is a more relevant prognostic factor compared with the CSS rate, especially for the survivors of poor prognosis cancers.

One of the advantages of the CS model compared with the Kaplan–Meier survival curve or multivariate regression models is that it offers more intuitive and quantitative information about the cure rate. Even without information about cancer recurrence or progression, the percentage of remaining patients when the CS rate reaches to a plateau (e.g., > 90%) could be used as a surrogate for the stable cure rate of the disease [14]. By using CS analysis, the benefit of treatments can also be evaluated; the difference of the percentage of patients who reach a plateau of CS rate between control and treatment groups can be considered the improved cure rate from the treatment [19–24].

In the present study, multivariate analyses of cancer-specific CS rates at 1, 3, and 5 years after diagnosis also provided useful information that T and M categories were the most significant prognostic factors even at 5 years after diagnosis, whereas surgery and radiotherapy were not. This finding suggests that patients with high risks might benefit from adjuvant treatments including systemic therapy after initial radical treatments.

There is no argument based on our analysis that complete resection with negative margin is a necessary condition for potentially curative treatment, whereas the role of both chemotherapy and radiotherapy have not been fully established [25–27]. In our present study, surgery increased the 5-year CSS rate to 28.5% with a 3-year cancer-specific CS rate of 90.5% at 5 years after diagnosis. However, radiotherapy did not increase CSS or CS rates at 5 years after diagnosis even after covariate adjustment. In the subgroup analysis of the surgery group, although radiotherapy increased the 1-year CSS rate, the lower cancer-specific CS rate at 1 year after diagnosis compared with the non-radiotherapy group indicated a poor prognosis at late period, resulting in no difference in the CSS rate at 5 years after diagnosis between the radiotherapy and non-radiotherapy groups.

Hyder et al. [28] demonstrated a similar conclusion in a propensity score-matched SEER data analysis that adjuvant external beam radiotherapy after curative-intent resection for GB cancer showed a survival benefit at 1 year after diagnosis and the benefit dissipated at 5 years after diagnosis. This result gives an impression that although radiotherapy may delay the progression of the disease, it can not increase long-term overall survival rate.

However, these results should be interpreted with caution. The discordance between the increased CSS rate and decreased cancer-specific CS rate at 1 year after diagnosis implies survivorship bias; patients who have benefit from radiotherapy might be those at high risk. In spite of the survival benefit of radiotherapy, the survival of the patients at high risk may be shorter than that of the patients at low risk, resulting in the change of the patient cohorts in the late period. Even in the study of propensity score-matched analysis of Hyder et al. [28], the patients with lymph node involvement had a long-term survival benefit from radiotherapy. Wang et al. [5, 29] built nomograms from the SEER database to predict the individualized survival benefit of adjuvant radiotherapy or chemoradiotherapy for patients with resected GB cancer, and these nomograms indicated that as least patients with T2 or N1 disease will gain survival benefit from radiotherapy.

One possible limitation of this study is that the consecutively decreasing number of patients in these time series multivariate analyses might have an influence on statistical significance. However, the sample size at 5 years after diagnosis was more than 600 for the analysis of the five variables, suggesting the impact would be minor.

If most of the patients with a specific adverse feature died during the early period, the cancer-specific CS rate would be less meaningful. For example, the estimated 1-year survival rate of patients in the non-surgery group was only 25.2%. In that case, a high cancer-specific CS rate at 5 years after diagnosis cannot be translated automatically into useless value of surgical treatment. Moreover, the Cox hazard ratio regression at late period is less powerful because of an insufficiently large sample size of patients at high risk (e.g., those with N1-2 diseases). A small number of survivors at 5 years after diagnosis reflects the low 5-year CSS rate in a risk group, whereas the statistical power of the cancer-specific CS rate at 5 years after diagnosis might be weakened by this small sample size at 5 years after diagnosis.

Jaundice and abnormal liver function could affect the patients’ prognosis, and the presence of gallstone(s) is also a major risk factor for GB cancer [30–32]. However, these statuses were not included in the SEER database.

SEER data did not offer any information on tumor recurrence or progression, surgical resection margin, and chemotherapy. With the data of patients with tumor recurrence, the conditional progression-free survival rate could be calculated and this rate would be more appropriate to serve as a surrogate for the cure rate of the patients with GB cancer [33].

## Conclusions

The cancer-specific CS rate of patients with GB cancer offers more accurate survival information compared with the CSS rate to patients who survived for a certain period. T and M categories were still significant prognostic factors even 5 years after diagnosis, whereas local treatments at the time of diagnosis were not, suggesting that further adjuvant treatments might be helpful for the patients with high T and M categories.
